# Trends in tubal sterilization and territorial disparities in Brazil: a nationwide time series study from 2013–2022

**DOI:** 10.61622/rbgo/2026rbgo69

**Published:** 2026-09-23

**Authors:** Ana Luiza Nepomuceno Sampaio, Alessandro Vidal de Oliveira, Jonathas Adriel Tavares Amaral, Rui Wanderley Mascarenhas

**Affiliations:** 1 Universidade do Estado do Pará Faculdade de Medicina Belém PA Brazil Faculdade de Medicina, Universidade do Estado do Pará, Belém, PA, Brazil.; 2 Universidade do Estado do Pará Laboratório de Cirurgia Experimental Belém PA Brazil Laboratório de Cirurgia Experimental, Universidade do Estado do Pará, Belém, PA, Brazil.; 3 Fundação Hospital de Clínicas Gaspar Vianna Departamento de Urologia Belém PA Brazil Departamento de Urologia, Fundação Hospital de Clínicas Gaspar Vianna, Belém, PA, Brazil.

**Keywords:** Sterilization, tubal, Female sterilization, Contraception, Epidemiologic studies, Brazil

## Abstract

**Objective:**

To analyse the trends in tubal sterilization in Brazil, regions and Federative Units by age group, race/skin colour and type of procedure from 2013 to 2022.

**Methods:**

Time series study with data from the Hospital Information System with females aged 20–59 years who underwent tubal sterilization procedures from 2013 to 2022 and defined by the standardized code utilized in the Brazilian Unified Health System for gathering data. Trends in tubal sterilization rates by region, age group, race/skin colour and procedure type were assessed. We estimated the average annual percent change (AAPC) and the 95% confidence interval (CI) of the tubal sterilization rates by Joinpoint regression. We compared the trends by coincidence test.

**Results:**

There were 719,426 tubal sterilizations in Brazil, with an overall upward trend (AAPC: 4.3; CI: 1.0–7.8). However, trends remained stationary in the Southeast (AAPC: 2.6; CI: -0.2–5.5) and South (AAPC: 0.4; CI: -3.5–4.4). All age groups had upward trends, except those aged 50–54 (AAPC: 6.2; CI: -7.5–22.0) and 55–59 years (AAPC: 7.4; CI: -4.9–21.4), which showed stationary trends. The analysis by race/skin colour predominantly indicated upward trends with greater AAPC amongst Browns. There were upward trends for procedures performed non-postpartum (AAPC: 8.5; CI: 7.5–9.4) and stationary trends for those performed postpartum (AAPC: 1.0; CI: -5.2–7.7).

**Conclusions:**

While Brazil as a whole exhibited upward trend over the period, significant disparities were observed across regions and populations, reflecting structural inequalities in access and choice of contraception methods.

## Introduction

Unintended pregnancy rates in Latin America were almost twice as those in Europe and North America between 2015–2019, with half resulting in abortion.^(1)^ The tubal sterilization (TS) is a definitive contraceptive method due to occlusion of section of the uterine tubes during labour or electively.^(2)^ The 15–45 age group are suitable candidates for this procedure, as fertility declines with age due to a reduction in the number and quality of ovarian follicles, in addition to decrease uterine capacity to sustain pregnancy.^(3)^

TS offers a favourable cost-benefit ratio for couples not wishing to have children, with an annual failure rate of only 0.17%, surpassing other contraceptive methods.^(4)^ However, post-procedure regret may occur, and reversal is not always viable.^(5)^ Understanding that the decision to undergo TS encompass multiple aspects increases the knowledge of population and their social determinants.^(6)^ Global prevalence of TS varies,^(2,7-9)^ being the most common contraceptive method in developing countries.^(7)^ Nevertheless, modern contraceptive methods surpass the TS^(8)^ highlighting substantial differences in contraception access, mostly in populations at risk.^(10)^ National literature lacks studies on TS trends,^(2)^ which are essential to reduce unintended pregnancies and unsafe abortions,^(11,12)^ in addition to broadening family planning services.^(13)^

The equitable provision of contraceptive methods across Brazil faces significant challenges due to economic constraints, logistical barriers, and constitutional mandate of universality under the Unified Health System (SUS). This scenario is particularly relevant as more than 70% of the Brazilian population relies exclusively on the SUS for healthcare access.^(14)^ Moreover, analysing periods that encompass moments of regulatory transition – such as the years leading up to the implementation of Law No. 14,443 in Brazil, which revised sterilization eligibility criteria – is essential to understand how policy changes may influence future trends.^(15,16)^

Therefore, we aimed to analyse the trends in TS in Brazil and its regions and Federative Units (FUs) according to age group, race/skin colour, and type of procedure from 2013–2022.

## Methods

### Study design and setting

This is a time series study of TS in Brazil from 2013–2022 according to age group, race/skin colour, and type of procedure with data from the Hospital Information System of Brazilian Unified Health System (SIH/SUS).^(17)^ Brazil is a country located in South America and is divided in five regions which are constituted by 26 FUs and a federal district. Currently, Brazilian population is estimated to be 203 million inhabitants, of which 51.5% are female and about 61 million of the female population are in reproductive age.^(18)^

### Data sources

Data on TS were collected from the SIH/SUS in June 2024 by using the *microdatasus* package^(19)^ developed for R v4.1.2 software. The demographic data were collected from the Department of Technology and Information of the Brazilian Unified Health System and the Brazilian Institute of Geography and Statistics.^(20)^

### Study population and variables

We analysed all TS available in SIH/SUS from 2013–2022 performed on females aged 20–59 years at the time of the procedure. We used the standardised procedure codes in the SIH/SUS database instead of the ICD-10 codes for gathering data on non-postpartum TS (0409060186) and postpartum TS (04110100420). The demographic data were aggregated according to region and FU of residence, age group, and race/skin colour.^(20,21)^ We calculated the TS rates (TSR) per 100,000 inhabitants, dividing the annual number of procedures by the female population. The following variables were analysed:

1. Age group (in years): 20–24, 25–29, 30–34, 35–39, 40–44, 45–49, 50–54, and 55–59;
2. Race/skin colour: White, Black, and Brown;
3. FU of residence: Acre, Alagoas, Amapá, Amazonas, Bahia, Ceará, Distrito Federal, Espírito Santo, Goiás, Maranhão, Mato Grosso, Mato Grosso do Sul, Minas Gerais, Pará, Paraíba, Paraná, Pernambuco, Piauí, Rio de Janeiro, Rio Grande do Norte, Rio Grande do Sul, Rondônia, Roraima, Santa Catarina, São Paulo, Sergipe, and Tocantins;
4. Region of residence: North, Northeast, Southeast, South, and Midwest;
5. Type of procedure: postpartum (04110100420) and non-postpartum (0409060186).

### Statistical analysis

We estimated the annual percent change (APC), the average annual percent change (AAPC) and the 95% confidence intervals (CI) of the TSRs via Joinpoint regression^(22)^ to understand whether multiple line segments better describe the data than a single line segment. Join points or inflection points were estimated by Monte Carlo permutation and the models were compared according to the lowest Bayesian information criterion.^(22)^ Moreover, trends in FUs were compared by pairwise coincidence test to identify disparities,^(23)^ for which the data was segregated according to the following variables: age group (20–39 years vs. 40–59 years), race/skin colour (Whites vs. Blacks and Browns), type of procedure (postpartum vs. non-postpartum). The pairwise test compared whether regression models were identical to a single intercept and slope in the FU data.^(23)^

Trends were classified as upward, when the APC or AAPC were positive with a positive CI; downward, when the APC or AAPC were negative with a negative CI; and stationary, when CIs encompassed the zero, regardless of the APC or AAPC values. The pairwise comparisons were classified as non-coincident when p < 0.05. Additionally, we estimated the annual frequency of TSR together with their percentages, the average annual age of females that underwent TS together with their standard-deviations (SD).

We used Joinpoint software v4.9.1.0 with log-linear data transformation and CI estimated by parametric method with 4,499 permutations. The year was the independent variable and the TSRs were the dependent variable. The models produced did not account for autocorrelation as it could lower statistical power to detect join points;^(22)^ however, models accounted for heteroscedasticity by standard error calculations. All analyses were performed with two-tailed tests and p < 0.05 as a threshold.

### Ethical aspects

This study was conducted in a public database with disidentified data and did not need ethical approval, which is in accordance with international guidelines.

## Results

### TS characteristics

There were 719,426 TS in Brazil, of which 50.4% were performed postpartum. The average age of females who underwent postpartum TS non-postpartum was 32 years (SD: 5.2), while the average age amongst those who underwent postpartum was 31.6 years (SD: 5.3). Most TS were performed on the 30–34 age group (32.3%) and amongst Browns (57.6%), with a greater number of procedures in the Southeast (45.1%). Additional data are in table 1.

**Table 1:** Characteristics of tubal sterilizations rates by region of residence, Federative Unit, age group, race/skin colour and type of procedure

Variables			Year of procedure									
			2013	2014	2015	2016	2017	2018	2019	2020	2021	2022
Mean age (years) (SD)			31.4(5.20)	31.4(5.20)	31.6(5.20)	31.7(5.20)	31.8(5.02)	31.9(5.20)	31.9(5.20)	31.8(5.30)	31.9(5.30)	32.0(5.30)
No. of tubal sterilizations			58,555	63,705	61,019	63,265	66,963	77,626	84,624	66,450	70,868	106,351
By region of residence (%)												
	North		3,719(6.35)	4,041(6.34)	3,864(6.00)	4,371(6.91)	5,099(7.61)	6,436(8.29)	7,250(8.57)	6,738(10.14)	7,530(10.630)	11,011(10.35)
		Rondônia	893(24.01)	1,336(33.06)	1,605(41.54)	1,766(40.40)	1,932(37.89)	1,840(28.59)	1,900(26.21)	1,467(21.77)	1,328(17.64)	1,985(18.03)
		Acre	65(1.75)	89(2.20)	50(1.29)	69(1.58)	140(2.75)	115(1.79)	161(2.22)	196(2.91)	118(1.57)	197(1.79)
		Amazonas	275(7.39)	211(5.22)	287(7.43)	526(12.03)	564(11.06)	809(12.57)	865(11.93)	1,038(15.41)	1,257(16.69)	2,017(18.32)
		Roraima	547(14.71)	468(11.58)	329(8.51)	294(6.73)	518(10.16)	469(7.29)	558(7.70)	425(6.31)	366(4.86)	607(5.51)
		Pará	960(25.81)	990(24.50)	894(23.14)	1,053(24.09)	1,211(23.75)	2,204(34.24)	2,257(31.13)	2,154(31.97)	2,952(39.20)	3,641(33.07)
		Amapá	0(0)	0(0)	0(0)	0(0)	1(0.02)	225(3.50)	485(6.69)	387(5.74)	433(5.75)	652(5.92)
		Tocantins	979(26.23)	947(23.43)	699(18.09)	663(15.17)	733(14.38)	774(12.03)	1,024(14.12)	1,071(15.89)	1,076(14.29)	1,912(17.36)
	Northeast		13,093(22.36)	15,373(24.13)	14,017(22.98)	15,853(25.06)	14,605(21.81)	19,409(25.00)	20,971(24.78)	16,857(25.37)	17,670(24.94)	31,354(29.49)
		Maranhão	803(6.13)	422(2.75)	460(3.28)	613(3.87)	1,063(7.28)	2,059(10.61)	2,194(10.46)	2,278(13.51)	2,511(14.21)	3,567(11.38)
		Piauí	362(2.76)	318(2.07)	468(3.34)	537(3.39)	483(3.31)	564(2.91)	567(2.70)	199(1.18)	167(0.94)	503(1.60)
		Ceará	3,112(23.77)	3,773(24.54)	3,177(22.66)	3,405(21.48)	3,447(23.61)	4,001(20.62)	4,220(20.13)	3,729(22.13)	4,118(23.31)	4,848(15.46)
		Rio Grande do Norte	775(5.92)	837(5.44)	797(5.69)	588(3.71)	662(4.53)	693(3.57)	683(3.26)	503(2.98)	814(4.60)	1,072(3.42)
		Paraíba	1,595(12.18)	1,504(9.79)	1,625(11.59)	1,731(10.92)	1,497(10.25)	1,416(7.30)	2,075(9.89)	1,496(8.88)	1,459(8.26)	2,230(7.11)
		Pernambuco	1,452(11.09)	1,872(12.18)	1,793(12.79)	2,355(14.85)	2,295(15.71)	2,918(15.04)	3,492(16.65)	3,027(17.95)	2,693(15.23)	4,441(14.16)
		Alagoas	13(0.10)	12(0.08)	25(0.18)	40(0.25)	55(0.38)	55(0.28)	46(0.22)	90(0.53)	192(1.09)	452(1.44)
		Sergipe	1,498(11.44)	1,950(12.68)	1,554(11.08)	1,488(9.39)	960(6.57)	1,055(5.44)	1,042(4.97)	515(3.06)	772(4.37)	1,512(4.82)
		Bahia	3,483(26.61)	4,685(30.47)	4,118(29.39)	5,096(32.14)	4,143(28.37)	6,648(34.25)	6,652(31.73)	5,020(29.78)	4,944(27.99)	12,729(40.61)
	Southeast		28,122(48.03)	30,496(47.88)	29,224(47.91)	28,850(45.59)	32,083(47.91)	35,098(45.22)	38,206(45.16)	29,334(44.14)	31,051(43.79)	41,906(39.40)
		Minas Gerais	7,775(27.64)	9,642(31.62)	9,537(32.63)	8,414(29.17)	9,055(28.22)	9,854(28.07)	10,013(26.21)	7,175(24.46)	6,840(22.03)	12,022(28.69)
		Espírito Santo	1,001(3.56)	581(1.91)	644(2.20)	751(2.60)	698(2.18)	724(2.06)	882(2.31)	572(1.95)	685(2.21)	689(1.64)
		Rio de Janeiro	1,788(6.36)	2,121(6.96)	2,254(7.71)	2,411(8.36)	3,493(10.89)	4,328(12.33)	5,185(13.57)	4,860(16.57)	5,523(17.78)	6,907(16.48)
		São Paulo	17,558(62.44)	18,152(59.51)	16,789(57.46)	17,274(59.87)	18,837(58.71)	20,192(57.54)	22,126(57.91)	16,727(57.02)	18,003(57.98)	22,288(53.19)
	South		8,208(14.02)	8,093(12.70)	7,784(12.76)	8,126(12.84)	8,365(12.49)	8,956(11.54)	9,413(11.12)	6,297(9.48)	6,654(9.39)	10,532(9.91)
		Paraná	3,312(40.35)	3,438(42.48)	3,230(41.48)	3,398(41.82)	3,894(46.54)	4,261(47.58)	4,485(47.65)	3,155(50.09)	3,160(47.49)	5,117(48.58)
		Santa Catarina	1,758(21.42)	1,905(23.54)	1,883(24.18)	1,876(23.08)	1,892(22.61)	2,155(24.06)	2,443(25.95)	1,660(26.36)	1,820(27.34)	2,779(26.38)
		Rio Grande do Sul	3,138(38.23)	2,750(33.98)	2,671(34.30)	2,852(35.10)	2,579(30.84)	2,540(28.36)	2,485(26.40)	1,482(23.54)	1,674(25.17)	2,636(25.03)
	Midwest		5,413(9.24)	5,702(8.95)	6,130(10.05)	6,065(9.58)	6,811(10.17)	7,727(9.96)	8,784(10.38)	7,224(10.87)	7,963(11.23)	11,548(10.86)
		Mato Grosso do Sul	1,272(23.50)	1,285(22.54)	1,419(23.15)	1,354(22.32)	1,627(23.89)	1,644(21.27)	1,835(20.89)	1,266(17.53)	1,405(17.64)	2,143(18.56)
		Mato Grosso	1,562(28.86)	1,708(29.95)	1,689(27.56)	1,820(30.01)	1,931(28.35)	2,527(32.71)	2,919(33.24)	2,333(32.30)	2,815(35.34)	3,994(34.60)
		Goiás	2,146(39.64)	2,114(37.07)	2,539(41.43)	2,506(41.32)	2,848(41.81)	3,081(39.86)	3,414(38.87)	3,017(41.77)	3,012(37.82)	4,739(41.04)
		Distrito Federal	433(8.00)	595(10.44)	483(7.88)	385(6.35)	405(5.95)	475(6.15)	616(7.02)	608(8.41)	731(9.18)	672(5.82)
By age group (in years) (%)												
	20–24		4,033(6.89)	4,237(6.65)	4,006(6.57)	3,727(5.89)	3,929(5.87)	4,379(5.64)	4,894(5.78)	4,487(6.75)	4,532(6.39)	5,969(5.61)
	25–29		19,137(32.68)	20,932(35.75)	19,642(33.54)	20,029(34.21)	20,560(35.11)	23,259(29.96)	25,322(29.92)	20,040(30.16)	21,395(30.19)	32,238(30.31)
	30–34		19,067(32.56)	20,631(35.23)	19,688(33.62)	20,668(35.30)	21,587(36.87)	25,454(32.79)	27,718(32.75)	21,154(31.83)	22,478(31.72)	33,644(31.63)
	35–39		11,981(20.46)	13,116(22.40)	12,929(22.08)	13,870(23.69)	15,353(26.22)	18,010(23.20)	19,408(22.93)	15,001(22.57)	16,170(22.82)	24,502(23.04)
	40–44		3,918(6.69)	4,305(7.35)	4,333(7.40)	4,535(7.74)	5,054(8.63)	5,979(7.70)	6,668(7.88)	5,317(8.00)	5,771(8.14)	9,056(8.52)
	45–49		390(0.67)	452(0.77)	402(0.69)	415(0.71)	457(0.78)	510(0.66)	588(0.69)	427(0.64)	485(0.68)	881(0.83)
	50–54		24(0.04)	20(0.03)	15(0.03)	18(0.03)	18(0.03)	28(0.04)	17(0.02)	20(0.03)	26(0.04)	42(0.04)
	55–59		5(0.01)	12(0.02)	4(0.01)	3(0.01)	5(0.01)	7(0.01)	9(0.01)	4(0.01)	11(0.02)	19(0.02)
By race/skin colour (%)												
	Whites		17,752(30.50)	18,712(29.57)	17,509(29.15)	18,011(28.27)	20,100(30.79)	21,563(28.60)	21,841(26.61)	16,785(26.01)	16,599(23.83)	23,380(22.34)
	Blacks		2,395(4.11)	2,642(4.18)	2,623(4.37)	2,757(4.48)	3,200(4.90)	3,770(5.00)	4,132(5.03)	3,431(5.32)	3,541(5.08)	5,087(4.86)
	Browns		21,751(37.37)	24,695(39.03)	23,492(39.10)	23,911(38.86)	25,962(39.77)	30,948(41.05)	37,232(45.36)	29,852(46.26)	33,132(47.57)	55,221(52.76)
	Ignored		16,309(28.02)	17,223(27.22)	16,451(27.38)	16,854(27.39)	16,016(24.54)	19,110(25.35)	18,876(23.00)	14,459(22.41)	16,372(23.51)	20,970(20.04)
By type of procedure (%)												
	Postpartum		23,593(40.29)	26,326(41.32)	27,269(44.69)	28,477(45.01)	33,663(52.63)	37,738(48.62)	42,255(49.93)	43,570(65.57)	47,398(66.88)	52,057(48.95)
	Non-postpartum		34,962(59.71)	37,379(58.68)	33,750(55.31)	34,788(54.99)	33,300(52.06)	39,888(51.38)	42,369(50.07)	22,880(34.43)	23,470(33.12)	54,294(51.05)

Abbreviations: SD, standard deviation

### Trends in Brazil and its regions

The trends in TS varied according to the Brazilian territory. Brazil as a unit had an upward trend (AAPC: 4.3, CI 1.0–7.8) despite the Southeast (AAPC: 2.6, CI -0.2–5.5) and South (AAPC: 0.4, CI -3.5–4.4) showing stationary trends. The North (AAPC: 10.2, CI 7.0–13.6), Northeast (AAPC: 6.8, CI 2.1–11.8), and Midwest (AAPC: 6.1, CI 3.0–9.3) had increasing trends (Table 2). The FUs that had the greater AAPCs were Alagoas (AAPC: 43.0, CI 26.2–61.9), Amazonas (AAPC: 23.5, CI 18.6–28.5), Maranhão (AAPC: 21.7, CI 13.2–30.9), Amapá (AAPC: 18.3, CI 0.1–39.9), Pará (AAPC 15.9, CI 11.2–20.7), and Rio de Janeiro (AAPC 15.7, CI 12.5–18.9), all of them with upward trends. Only Rio Grande do Sul (AAPC: -4.2, CI -8.3–[-0.1]) and Sergipe (AAPC: -7.2, CI -13.9–0.0) had downward trends (Table 2). Regarding age groups, only 50–54 (AAPC: 6.2, CI -7.5–22.0), and 55–59 years (AAPC: 7.4, CI -4.9–21.4) had stationary trends while all other age groups had upward trends (Table 2). Races/skin colours trends were mostly upward, with greater AAPC amongst Browns (AAPC: 8.6, CI 3.8–13.5), however, with stationary trends amongst Whites (AAPC: 1.8, CI -1.4–5.1). As for type of procedure, non-postpartum TS had an upward trend (AAPC: 8.5, CI 7.5–9.4) and postpartum TS had a stationary trend (AAPC: 1.0, CI -5.2–7.7) (Table 2).

**Table 2:** Trends of tubal sterilizations per 100,000 inhabitants by region of residence, Federative Unit, age group, race/skin colour, and type of procedure

Variable			Period	AAPC	95%CI	p-value	Trend
Region of residence							
Brazil			2013–2022	4.3	1–7.8	0.018	Upward
	North		2013–2022	10.2	7–13.6	<0.001	Upward
		Rondônia	2013–2022	1.8	-3.6–7.4	0.476	Stationary
		Acre	2013–2022	10.1	2.6–18.2	0.014	Upward
		Amazonas	2013–2022	23.5	18.6–28.5	<0.001	Upward
		Roraima	2013–2022	-3.2	-8.5–2.5	0.227	Stationary
		Pará	2013–2022	15.9	11.2–20.7	<0.001	Upward
		Amapá	2013–2022	18.3	0.1–39.9	0.049	Upward
		Tocantins	2013–2022	4.4	-4.1–13.5	0.322	Stationary
	Northeast		2013–2022	6.8	2.1–11.8	0.01	Upward
		Maranhão	2013–2022	21.7	13.2–30.9	<0.001	Upward
		Piauí	2013–2022	-0.7	-9.9–9.5	0.877	Stationary
		Ceará	2013–2022	2.6	0.4–4.9	0.025	Upward
		Rio Grande do Norte	2013–2022	1.9	-4.3–8.6	0.555	Stationary
		Paraíba	2013–2022	1.4	-2.6–5.4	0.456	Stationary
		Pernambuco	2013–2022	9.7	5.4–14.1	0.001	Upward
		Alagoas	2013–2022	43	26.2–61.9	0.001	Upward
		Sergipe	2013–2022	-7.2	-13.9–0	0.05	Downward
		Bahia	2013–2022	11	3.2–19.5	0.011	Upward
	Southeast		2013–2022	2.6	-0.2–5.5	0.066	Stationary
		Minas Gerais	2013–2022	0.6	-3.8–5.2	0.754	Stationary
		Espírito Santo	2013–2022	-2.9	-7.2–1.6	0.171	Stationary
		Rio de Janeiro	2013–2022	15.7	12.5–18.9	<0.001	Upward
		São Paulo	2013–2022	1.4	-1.1–3.9	0.248	Stationary
	South		2013–2022	0.4	-3.5–4.4	0.82	Stationary
		Paraná	2013–2022	2.6	-1.5–6.8	0.19	Stationary
		Santa Catarina	2013–2022	2.1	-1.7–6.1	0.234	Stationary
		Rio Grande do Sul	2013–2022	-4.2	-8.3–(-0.1)	0.047	Downward
	Midwest		2013–2022	6.1	3–9.3	0.002	Upward
		Mato Grosso do Sul	2013–2022	3	-0.9–7.1	0.117	Stationary
		Mato Grosso	2013–2022	8.9	5.4–12.6	<0.001	Upward
		Goiás	2013–2022	6.2	2.9–9.6	0.002	Upward
		Distrito Federal	2013–2022	3.5	-0.8–7.3	0.1	Stationary
Age group (in years)							
	20–24		2013–2022	3.8	1.2–6.5	0.01	Upward
	25–29		2013–2022	4.5	1.1–7.9	0.014	Upward
	30–34		2013–2022	4.9	1.4–8.5	0.012	Upward
	35–39		2013–2022	5.2	1.6–8.9	0.01	Upward
	40–44		2013–2022	5.9	2.2–9.7	0.006	Upward
	45–49		2013–2022	5.8	0.9–11	0.026	Upward
	50–54		2013–2022	6.2	-7.5–22	0.39	Stationary
	55–59		2013–2022	7.4	-4.9–21.4	0.214	Stationary
Race/skin colour							
	Whites		2013–2022	1.8	-1.4–5.1	0.24	Stationary
	Blacks		2013–2022	2.6	0.6–4.7	0.017	Upward
	Browns		2013–2022	8.6	3.8–13.5	0.003	Upward
Type of procedure							
	Postpartum		2013–2022	1	-5.2–7.7	0.72	Stationary
	Non-postpartum		2013–2022	8.5	7.5–9.4	<0.001	Upward

Abbreviations: AAPC, average annual percent change; 95%CI, 95% confidence interval

Figure 1 illustrates the temporal series of TSRs. The lowest TSR observed in Brazil as a unit was in 2013 (101.2/100,000 inhabitants) and the greatest was in 2022 (169.7/100,000 inhabitants). In all regions, the greatest TSRs were in 2022, especially in Midwest (231.1/100,000 inhabitants) and North (204.3/100,000 inhabitants) (Figure 1A). All age groups had their greatest TSRs in 2022, with 30–34 (197.4/100,000 inhabitants) and 25–29 (188.6/100,000 inhabitants) age groups had the highest values (Figure 1B). All races/skin colours had their greatest TSRs in 2022 (Whites: 86.6/100,000 inhabitants; Blacks: 80.0/100,000 inhabitants; Browns: 196.6/100,000 inhabitants) (Figure 1C). Both type of procedures had their greatest TSRs in 2022 (non-postpartum: 86.6/100,000 inhabitants; postpartum: 83.1/100,000 inhabitants), whereas the lowest TSR non-postpartum was in 2020 (39.6/100,000 inhabitants), the lowest postpartum TSR was in 2013 (40.8/100,000 inhabitants) (Figure 1D).

**Figure 1:**
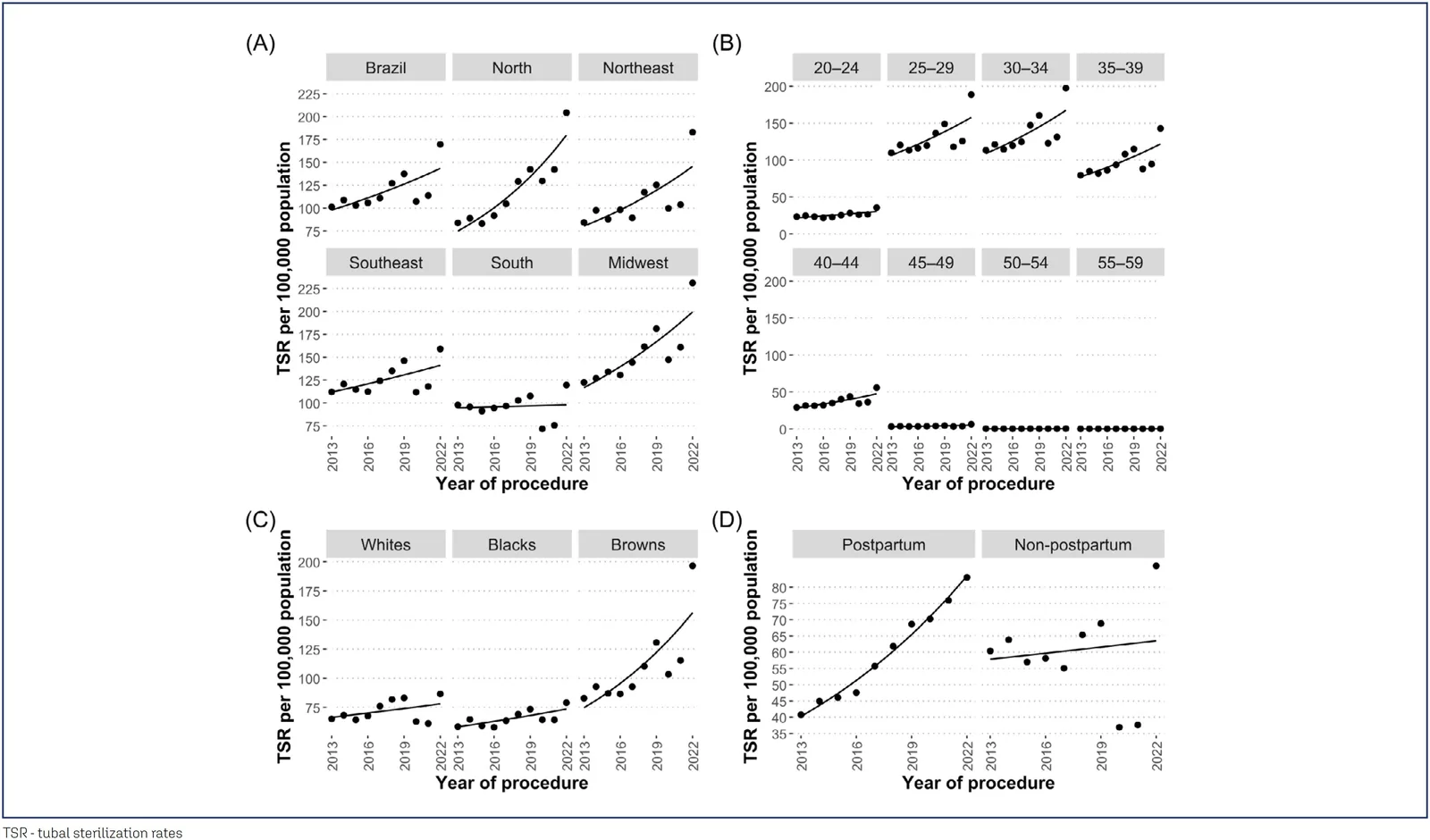
Time series trends of values observed and modelled by dots and lines, respectively, of tubal sterilization rates per 100,000 inhabitants per region of residence (A), age group in years (B), race/skin colour (C), and type of procedure (D).

TSR - tubal sterilization rates

We performed a post-hoc sensitivity analysis on the impact of including the 50–59 age group in the regional trends (Figure 2), in which a slight increase in the AAPC was seen in most of the regions when the 50–59 age group was excluded from the analyses. However, despite this slight increase in the AAPC, trends were not altered in Brazil as a unit (AAPC: 4.8, CI 1.4–8.3), North (AAPC: 11.1, CI 7.8–14.4), Northeast (AAPC: 7.5, CI 2.7–12.5), South (AAPC: 0.5, CI -3.3–4.5), and Midwest (AAPC: 6.6, CI 3.5–9.8). Only the Southeast displayed a different trend when excluding the 50–59 age group, with an AAPC of 3.1 (CI 0.2–6.0), characterising an increasing trend.

**Figure 2:**
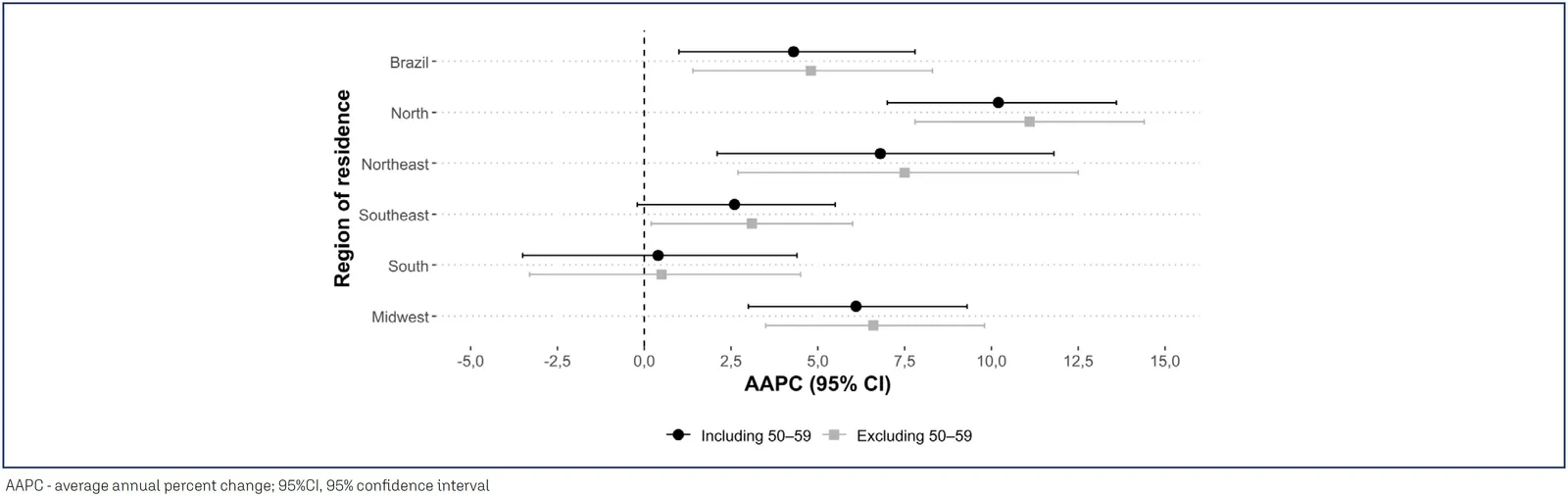
Sensitivity analysis to assess the influence of including the 50–59 age group on regional trends.

AAPC - average annual percent change; 95%CI, 95% confidence interval

### Disparities in Brazilian territory

Figure 3 displays the disparities in the trends found among the FUs analysed with pairwise coincidence test, in which the existence of non-coincident trends characterised the differences between observed trends. Within FUs, all of them had disparities in the age group analysis when comparing 20–39 years vs. 40–59 years, with higher AAPCs among the 40–59 population in comparison with those in the 20–39 age group; however, Alagoas, Paraná, and Rio de Janeiro had higher AAPCs among the 20–39 age group (Figure 3A). Regarding ethnic comparisons (Whites vs. Blacks and Browns), only Acre, Amapá, and Rio Grande do Sul had coincident trends, whereas all other FUs had non-coincident trends (Figure 3B). Regarding type of procedures (postpartum vs. non-postpartum), only Minas Gerais, Mato Grosso do Sul, Paraíba, Pernambuco, and Santa Catarina had coincident trends, while all other FUs had non-coincident trends, with higher AAPCs in the postpartum TS compared with non-postpartum TS in pairwise coincidence test (Figure 3C).

**Figure 3:**
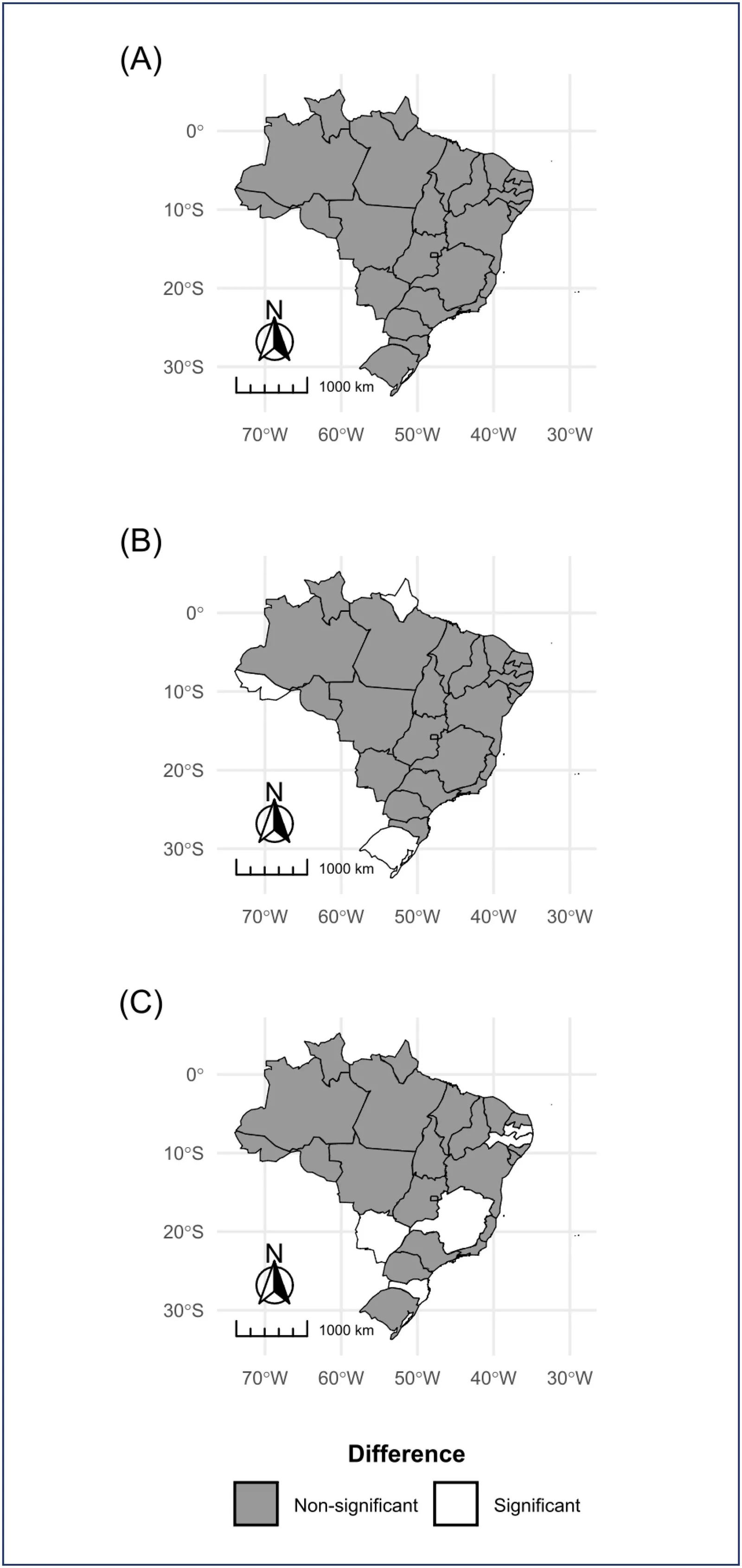
Pairwise comparison of trends in tubal sterilizations rates per 100,000 inhabitants across Brazilian Federative Units by age groups (A), racial groups (B), and types of procedure (C)

## Discussion

We found upward trends in TS in Brazil overall and in the North, Northeast, and Midwest regions. Most age groups showed increasing trends, except those aged 50–54 and 55–59 years, which remained stationary. According to race/skin colour, trends were predominantly upward, while White females presented stationary trends. Regarding the type of procedure, postpartum TS showed stationary trends, whereas non-postpartum procedures showed upward trends. Regional disparities were observed across all analysed variables.

TS in Brazil is frequently performed alongside caesarean section, possibly reflecting convenience as well as structural or legal factors influencing access.^(24)^ The higher prevalence observed among females aged 30–34 years reflects a pattern commonly reported in population-based studies and may be related to completed reproductive plans and lower rates of post-sterilization regret at this stage of life.^(5)^ This distribution is also consistent with evidence indicating that the biological reproductive span is concentrated between approximately 30.9 and 39.3 years.^(25)^ However, TS may be performed either before or after pregnancy, and its timing is influenced by individual preferences, clinical risk assessment, and access to healthcare services.^(26)^

Greater prevalence among Brown females may indicate socioeconomic disparities in the access to other contraceptive methods;^(27)^ though it may also relate to most population identifying as Brown. Higher prevalence in the Southeast may reflect demographic and structural characteristics of this region, including population concentration, and economic centralization, as described in national analyses.^(28)^

TS trends varied in Brazil, agreeing with studies performed in other countries. In India,^(9)^ the TSRs increased by 31.5% from 1992–2015, while in the United States (US),^(27)^ the trends kept stationary or downward. In Latin America, there was an upward trend, with a 25% prevalence in Brazil.^(2)^ Nevertheless, regional discrepancies remain, with higher TSRs and upward trends in North, Northeast, and Midwest, contrasting the stationary trends in South and Southeast. This stability is likely associated with expanded availability of alternative contraceptive methods, including options with benefits beyond contraception – such as menstrual regulation and non-contraceptive health benefits – possibly reducing reliance on permanent procedures, as observed in other countries^(7,29)^ and in Southern Brazil.^(10)^

Regional variations and differences between FU's may influence reproductive choices and reflect difference in healthcare organization.^(27)^ Declines or stability in TSRs have been associated with public policies that expand access to long-acting reversible contraception, introduce reproductive incentives, and strengthen primary healthcare services.^(7,30,31)^ Cultural factors and social norms also influence the acceptance of permanent contraceptive methods and shape reproductive choices.^(10,27,29,32)^ Evidence from multilevel analyses of contraceptive use in Brazil demonstrates substantial heterogeneity between FUs, with lower prevalence and odds of contraceptive use among females in several states of the North and Northeast – such as Alagoas, Maranhão, Amazonas, and Pará.^(33)^

Disparities among regions might reveal structural differences. A Brazilian study from 2012–2018 identified that the availability of family planning inputs in basic units is directly related to the Human Development Index.^(13)^ The North showed the worst indicators, with under 80% availability for all reversible methods except male condoms,^(13)^ reflecting precarious infrastructure and logistical challenges in less urbanized and dispersed areas,^(34)^ possibly explaining the higher TSRs found in the North.

Most TS occurred among females aged 25–39 years, consistent with previous literature.^(8,9,27,30)^ Sterilization is often sought as a definitive contraceptive method by females with higher parity and more structured family arrangements,^(9)^ which could explain the concentration of procedures in this age range. These findings suggest a possible influence of the age on this decision-making process, with females in intermediate age groups showing approximately twice the odds of undergoing TS, particularly among those who are married or previously married.^(27)^ At the same time, the higher AAPC observed among females aged 40–59 years may reflect a combination of delayed access to definitive contraception due to historical legal and organizational barriers and voluntary postponement of TS.^(35)^ In more educated and urban populations, prolonged use of reversible contraceptive methods and later reproductive decision-making have been associated with TS occurring at older ages, which may also contribute to the observed trends.^(9,10)^

We found a predominantly upward trend in race/skin colour analyses, especially among Browns. Previous evidence has shown higher odds of TS among Black females regardless of marital status, potentially reflecting disparities in reproductive access.^(27)^ Social vulnerability and territorial inequalities, marked by lower formal education, lower family income, poor housing conditions, lack of health insurance, and being Black or Brown may influence this pattern, possibly due to lower maintenance costs.^(11,30,36)^ These groups also receive more restrictive reproductive counselling and are more often encouraged to limit offspring compared to other ethnic and economic groups.^(27)^

Postpartum and non-postpartum TS shows divergences in our findings and in the literature.^(27,29,37-39)^ Postpartum TS is considered a safe and acceptable option when appropriately indicated, and demand for concurrent definitive contraception has been documented.^(37)^ In Brazil, however, before the enactment of sterilization law, several logistical barriers limited access to postpartum TS, including the need for scheduling a separate surgical procedure, longer waiting times, and restricted availability of surgical teams and hospital infrastructure.^(35)^ In this context, despite Brazil being the second country worldwide in caesarean section rates, postpartum TS remained legally restricted until 2022 unless medically indicated, which may have contributed to limited access and potential underreporting in medical records.^(38)^

The recent change in Brazilian law allowing TS during labour^(15)^ represents crucial progress in reproductive rights and independence. Previously, legal and bureaucratic barriers restricted access to permanent contraception.^(38)^ By aligning legislation with public needs, Brazilian government promotes a more humane and context-sensitive healthcare system.

Nonetheless, informed and voluntary consent remains essential, especially in sterilization procedures.^(13)^ In settings marked by gender inequality and restrictive norms, consent may be compromised, enabling pressure, misinformation, and coercive practices that distort individuals’ true reproductive intentions.^(13,34,39)^

Restrictive reproductive laws heighten vulnerability. In Latin America, unintended pregnancy rates are nearly double those seen in Europe or North America, with around half ending in induced abortion.^(1)^ The lack of safe options harms physical and mental health, imposes economic and social barriers, and raises the risk of intimate partner violence, further entrenching marginalisation.^(1)^

Universal access to reproductive healthcare is a public health priority and a strategy to reduce maternal and child mortality and poverty.^(12,27,34)^ However, stigma and limited access to legal abortion often lead to unsafe procedures, causing around 47,000 deaths and five million disabilities annually.^(35)^ In contrast, policies that promote education, support, and access to contraception enhance females’ well-being and contribute to more equitable societies.^(40,41)^

Our findings indicate important variations in TSRs over time, with an increase in 2022 across all Brazilian regions. The decline in previous years may be linked to the COVID-19 pandemic, which restricted healthcare services by reducing elective procedures in 2020 and 2021 due to system overload.^(42)^ Additionally, fear of attending healthcare facilities may have led females to postpone TS. The sharp rise in 2022 supports the hypothesis of suppressed demand, as individuals now regain access to the procedure.

This study is not free of limitations. Incomplete data, errors in data entry, underreporting, or limited adherence may be linked to secondary databases. Moreover, the SIH/SUS does not include private practice, thus, our findings primarily reflect trends among users of the SUS and may underestimate the real number of TS in Brazil. Brazilian diversity and heterogeneity were minimised by stratifying analyses by region, FU, age group, and race/skin colours, yielding more detailed results. The inclusion of populations aged 50–59 years old may limit the interpretation of our findings; however, due to the increased age of maternity worldwide and fertility rates in this age group,^(43)^ we opted to include these populations in our analyses to encompass all possibly fertile women, rather than including only the age groups with higher fertility rates. Additionally, only minor changes were seen in the AAPC when this population was excluded from analysis, indicating that despite their inclusion, trends were mostly unaltered, validating our findings.

Furthermore, due to the ecological nature of this study and lack of individual-level data, structural determinants for the increase or stability on TS trends were not analysed as variables in our study, precluding causal inference; thus, caution is needed when interpreting our findings. Despite these limitations, this is the first study to quantify TS trends in Brazil, offering essential insights into the country's contraception scenario, particularly following the 2022 sterilization law. Our findings provide a basis for comparison with other countries and future research, highlighting the need to expand access to contraceptive methods, supporting reproductive rights, and more equitable health policies.

## Conclusion

In conclusion, although Brazil as whole had an increasing trend in the period, there were significant disparities across all regions and populations, highlighting structural discrepancies in access and choices of contraceptive methods. Nonetheless, the trends in non-postpartum TS may indicate a change in reproductive choice patterns in Brazil, reinforcing the need of public policies that promote a more inclusive and informed approach on reproductive planning in all country.

## Data Availability

The research data are described in the article presented.
